# 759. Impact of *Clostridium difficile (CD*) Nucleic Acid Amplification Test (NAAT) Approval on Hospital-Onset *C. difficile* Infection (HO-CDI): A Diagnostic Stewardship Intervention

**DOI:** 10.1093/ofid/ofab466.956

**Published:** 2021-12-04

**Authors:** Francine Touzard-Romo, Gail Jackson, Sarah Andrea, Whitehead Valerie, Tiffany Chargualaf, John Lonks

**Affiliations:** 1 Newport Hospital, Alpert Medical School of Brown University, East Greenwich, Rhode Island; 2 Newport Hospital, Newport, Rhode Island; 3 Lifespan, Providence, Rhode Island; 4 The Miriam Hospital / Alpert Medical School of Brown University, Providence, Rhode Island

## Abstract

**Background:**

NAAT is highly sensitive in detecting toxigenic CD but if used inappropriately can lead to overdiagnosis and financial penalties. Despite diligent infection control (IC) measures, HO-CDI rates at our hospital remained above target benchmarks. We implemented mandatory CD testing approval to decrease HO-CDI rates.

**Methods:**

On 7/1/2019, we implemented CD testing approval for stool samples collected after admission day 3 in our 129-bed community hospital. An algorithm instructed providers about approval granted by IC 7 days-a-week. The micro-lab would not process samples unless pre-approved. We prospectively collected data on demographics, ICU, laxative, antibiotic use, CDI signs/symptoms, prior CDI and outcomes (length of stay, in-hospital death) and estimated unadjusted relative risk ratios comparing those whose test was approved vs not approved. We also performed an interrupted time series analysis to assess the trend change of CD testing and HO-CDI per 1000 patient days (x 1000-PD) in the 18 months following the intervention (7/2019 - 12/2020) compared to the pre-intervention period (01/2018 - 6/2019). Lastly, using the National Healthcare Safety Network criteria, we calculated pre and post-intervention Standard Infection Ratios (SIR).

**Results:**

A total of 72 samples required CD testing authorization; 65 (90%) were approved. Baseline demographics, in-hospital death and length of stay were similar in both groups, but approved patients were 4 times as likely to have ≥ 3 loose stools in 24h compared to not approved. The number of CD tests was 13 at baseline with a decrease of 6 tests in the 1st month of intervention (95% CI: -10.0, - 1.35), followed by an insignificant decline in the monthly trend (-0.14; 95%CI: -0.49, 0.20). There were 22 HO-CDI pre-intervention and 10 post-intervention. Pre-intervention, incidence of HO-CDI was 0.51 cases x 1000-PD and increased every month by 0.11 (95% CI: 0.07,0.16). In July 2019, there was a significant decline of 1.16 case x 1000-PD (95% CI: -1.99, -0.33), followed by monthly decline (-0.16; 95% CI: -0.23, -0.09). Our calculated SIR after the intervention decreased to 0.77 from 1.03.

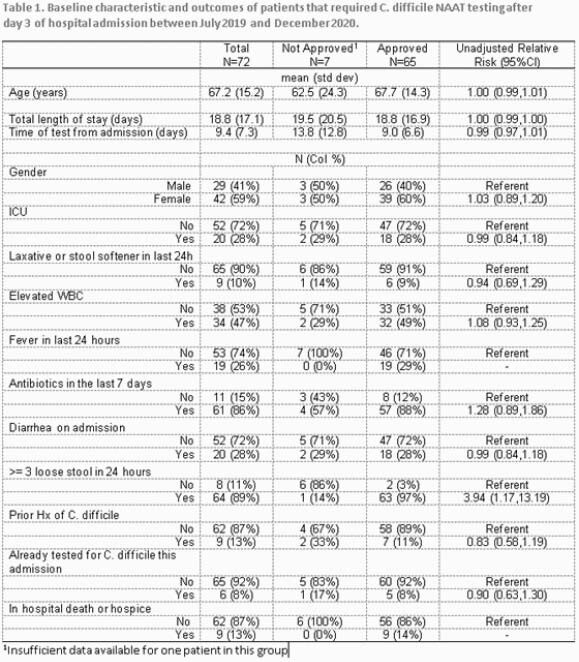

Trends of CD testing and HO-CDI in the pre-intervention and post-intervention period

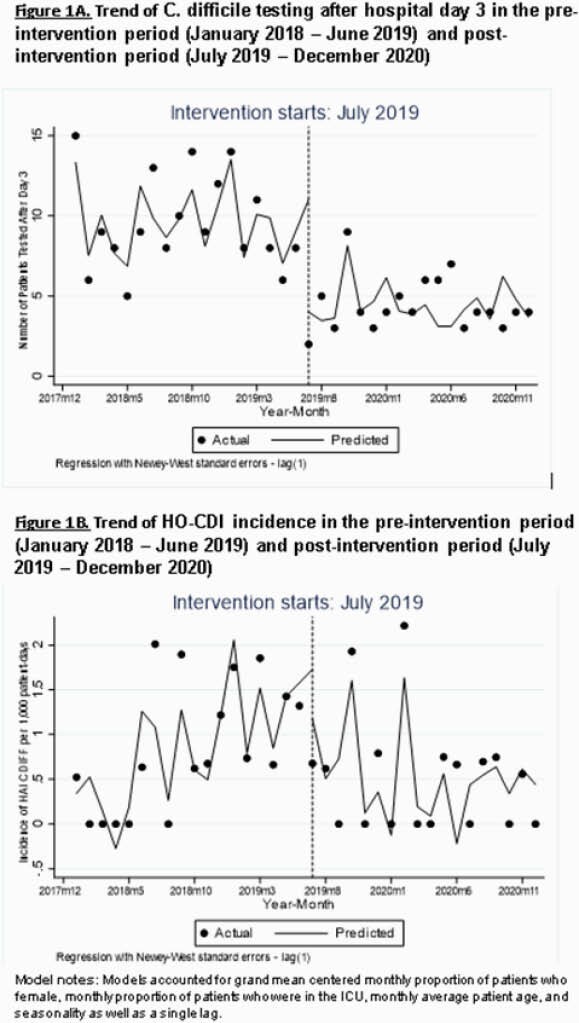

**Conclusion:**

CD testing approval is a successful strategy to optimize testing and lower HO-CDI rates, without resulting in worst outcomes even when CD test was not approved.

**Disclosures:**

**All Authors**: No reported disclosures

